# A Sustainable Solution to Skin Diseases: Ecofriendly Transdermal Patches

**DOI:** 10.3390/pharmaceutics15020579

**Published:** 2023-02-08

**Authors:** Eylul Gulsen Yilmaz, Emre Ece, Özgecan Erdem, Ismail Eş, Fatih Inci

**Affiliations:** 1 UNAM—National Nanotechnology Research Center, Bilkent University, Ankara 06800, Turkey; 2 Institute of Materials Science and Nanotechnology, Bilkent University, Ankara 06800, Turkey

**Keywords:** skin disease, transdermal patches, microneedles, controlled drug release, green biomaterials

## Abstract

Skin is the largest epithelial surface of the human body, with a surface area of 2 m^2^ for the average adult human. Being an external organ, it is susceptible to more than 3000 potential skin diseases, including injury, inflammation, microbial and viral infections, and skin cancer. Due to its nature, it offers a large accessible site for administrating several medications against these diseases. The dermal and transdermal delivery of such medications are often ensured by utilizing dermal/transdermal patches or microneedles made of biocompatible and biodegradable materials. These tools provide controlled delivery of drugs to the site of action in a rapid and therapeutically effective manner with enhanced diffusivity and minimal side effects. Regrettably, they are usually fabricated using synthetic materials with possible harmful environmental effects. Manufacturing such tools using green synthesis routes and raw materials is hence essential for both ecological and economic sustainability. In this review, natural materials including chitosan/chitin, alginate, keratin, gelatin, cellulose, hyaluronic acid, pectin, and collagen utilized in designing ecofriendly patches will be explored. Their implementation in wound healing, skin cancer, inflammations, and infections will be discussed, and the significance of these studies will be evaluated with future perspectives.

## 1. Introduction

**Figure 1:**
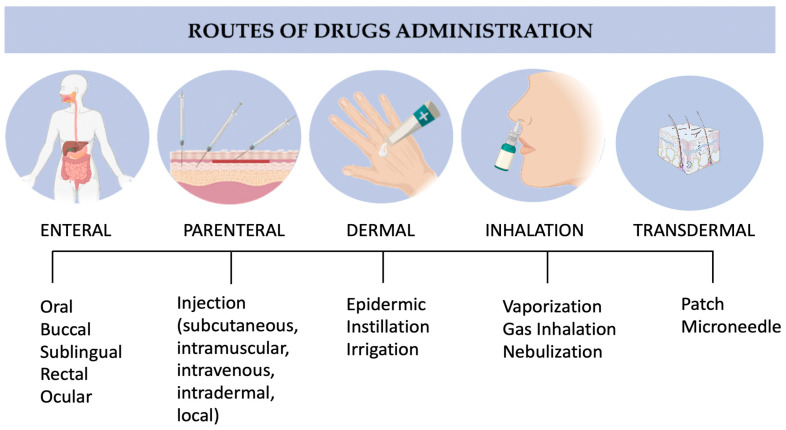
Main drug administration routes. Copyright permission for reuse from the refs. [9,10].

Being an external organ with the largest epithelial surface, skin is susceptible to several environmental factors, including microorganisms and hazardous chemicals [1,2]. On that account, skin diseases are one of the primary causes of global disease burden, impacting millions of people’s lives worldwide [3]. More than 3000 identified varieties of skin diseases, from eczema to skin cancer, have been reported to be experienced by patients [4]. Appropriate treatment of skin diseases includes either systemic (i.e., oral, sublingual, buccal, subcutaneous, intramuscular, and intravenous, etc.) or topical delivery of drugs (Figure 1). Most drugs are rapidly metabolized in liver upon oral administration, resulting in a reduced bioavailability before reaching the target site [5]. This situation requires the optimum oral dosage to be much higher than when administered intravenously.

An alternative to oral delivery and hypodermic injections, drugs can be delivered using dermal and transdermal routes. Additionally, predictable and extendable duration of activity, eliminated gastrointestinal absorption, controlled and adjustable dosage, and enhanced patient compliance also make them excellent candidates for next-generation drug delivery [6]. On the other hand, patches may cause skin irritations, and external factors may prevent the patch from sticking to the skin. Not all drugs are suitable to be administered using this route since low permeability of skin may limit the penetration of certain drugs. Despite these disadvantages, such a drug delivery method has eventually made a significant contribution to medical practice for releasing drugs in a controlled and minimally invasive fashion to treat skin diseases [7] (Table 1). To date, numerous drugs have been delivered using a number of dermal and transdermal adhesive patches. Such patches usually encounter limitations in surpassing the resistance of the uppermost layer of skin “stratum corneum”. To overcome this, microneedles, which are micron-sized needles orientated on a patch, are integrated into these patches to speed up drug penetration [8]. Current tackle still includes that the type of drugs and their maximum dosage, which can be loaded in these patches, are limited. To overcome this obstacle, novel polymers have been designed and manufactured to further exploit the benefits of such technological tools and expand their relevance in the diverse field of drug delivery.

**Figure 2:**
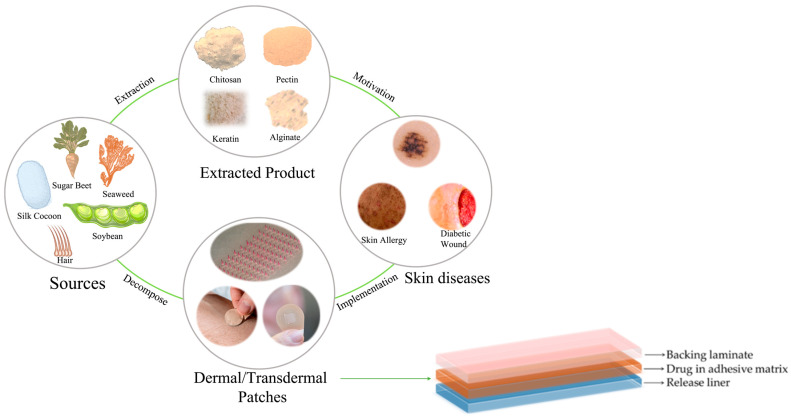
Dermal/transdermal patches are produced to treat different skin diseases using sustainable ecofriendly biopolymers derived from natural resources. Copyright permission for reuse from the refs. [33,34,35,36,37]. The image on the lower right corner illustrates the overall structure of a patch containing backing laminate, drug-loaded adhesive matrix, and release liner.

The major components of dermal/transdermal drug delivery systems comprise the drug molecule, a polymer complex, and adhesive material [22]. Briefly, polymers are critical materials for producing drug delivery patches; hence, polymer selection is a critical step in such processes since their properties determine the release of drugs at a required therapeutic rate. Advances in polymer science have paved the way for designing novel transdermal patches, which constitutes a rapidly increasing market projected to reach ~USD 87 billion by 2030 [23]. However, the current manufacturing route of such polymers typically involves synthetic processes. The key advantage of such processes is that polymers can be fabricated with desired mechanical and chemical assets; however, they are difficult to degrade naturally by biological processes, dramatically impacting the environment due to high amount of waste generation. Hence, the main focus on polymer synthesis has been directed toward ecofriendly or “green” production routes, which represent a sustainable alternative. These next-generation polymers are obtained from nonedible and highly accessible plants as well as agro-food and industrial by-products [24]. Despite minimized environmental impact, they may still show inferior performance regarding end-use applications compared to synthetic counterparts [25]. A wide range of natural polymers such as chitosan [26], cellulose [27], pectin [28], alginate [29,30], gelatin [31], and starch [32] have been utilized to manufacture ecofriendly patches to treat skin diseases (Table 2). The features such as swelling, biodegradability, stretchability, drug loading capacity, and drug release rate need to be taken into account for manufacturing patches for a specific skin disease (Figure 2).

Despite being a billion-dollar sector and new research being conducted, ecofriendly patch-mediated drug delivery technology is still in its infancy, and several issues remain to be addressed before it becomes mainstream. The goal of this review is to comprehensively present ecofriendly alternatives to synthetically produced polymeric patches, which hold a wide range of applications in treating skin-related diseases. These diseases are briefly addressed regarding their treatability via dermal and transdermal patches, which are described as well. Ecofriendly patches used in skin diseases are explored by discussing their types, manufacturing routes, characteristics, and applicability in medical practice considering the pros and cons. To conclude, we herein introduce the current status of ecofriendly patches in clinical applications to validate their efficacy against skin diseases.

## 2. Skin Diseases

**Figure 3:**
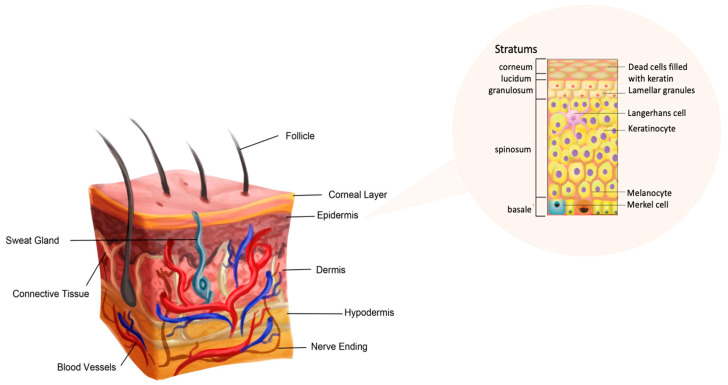
The structure of skin with all the characteristic of stratums. Copyright permission for the reuse from the refs. [44,45].

The skin is a complex and dynamic organ that is composed of several layers, each with its own exclusive characteristics (Figure 3) [44,45]. The epidermis is the outermost layer of a skin, and it is responsible for protecting the body from external factors. It is composed of multiple layers of cells that are constantly being shed and replaced. The stratum corneum, which serves as the outermost layer of the epidermis, is composed of dead skin cells that form a protective barrier [44,45]. Additionally, the stratum lucidum, found typically in thick skin such as the soles of the feet and palms of the hands, is a thin, transparent layer of the epidermis that helps to provide an additional barrier against external elements and friction. The stratum granulosum—acting as a transition layer—helps to protect the deeper layers of the skin. Furthermore, the stratum spinosum—a thicker layer—contains cells that are actively dividing [44,45]. Lastly, the stratum basale—the deepest layer of the epidermis—is responsible for the production of new skin cells. The dermis—the middle layer of the skin—is composed of connective tissue. It contains blood vessels, nerves, and sweat glands. The dermis is responsible for maintaining the elasticity and strength of skin [46]. The hypodermis—the innermost layer of the skin—is composed of fat and connective tissue, and it serves as insulation and cushioning for the body. The hypodermis also helps to regulate the body’s temperature. These three layers of skin work coherently to protect the body from external factors, regulate body temperature, and aid in sensation.

**Figure 4:**
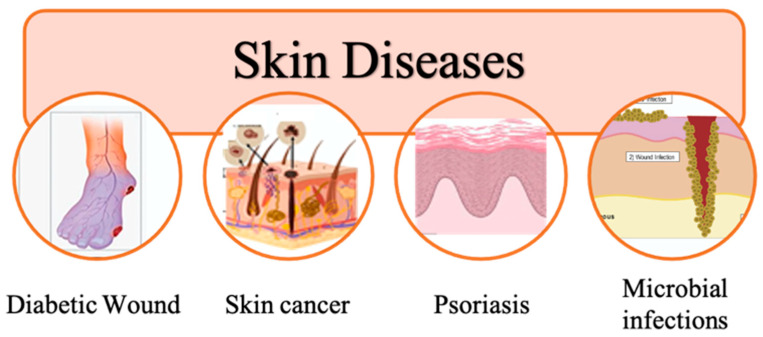
Changes in the skin caused by some of the most common skin diseases in humans. Copyright permission for reuse from the refs. [50,51,52,53].

The symptoms and degree of skin diseases vary extensively. They may be fleeting or long-lasting, painful or not. Several skin problems are minor, while the others pose a serious risk to life. In addition, skin diseases play an important role in the diagnosis and treatment of some internal diseases as well. Skin is not just an external organ, but it can also provide vital information about the overall health of an individual [46]. Skin diseases can be caused by a variety of internal factors such as hormonal imbalances, nutritional deficiencies, and underlying medical conditions [47]. For example, certain skin conditions such as acne, eczema, and psoriasis can be caused by, for instance, autoimmune disorders and inflammatory conditions [47]. Similarly, skin rashes and discoloration can be a symptom of other diseases such as diabetes and liver disease [48]. Therefore, skin diseases play an important role in the diagnosis and treatment of some internal diseases. By examining the skin as an organ, physicians would have valuable insights into the underlying causes of a patient’s symptoms. This knowledge can help to guide the diagnosis and treatment of the diseases, which in turn can improve the patient’s overall health and quality of life. In summary, skin diseases should not be overlooked or dismissed as only external issues, as they can also be a sign of underlying other diseases and play an important role in the diagnosis and treatment of these diseases [49]. There are a myriad of different skin diseases such as psoriasis, acne, rosacea, allergic diseases, diabetic wound, microbial infections, skin cancer, lupus, and so on (Figure 4) [49]. Among the reported lots of skin diseases, diabetic wound, skin cancer, psoriasis, and microbial infections are frequently used for transdermal skin patches.

### 2.1. Diabetic Wound

Diabetes mellitus (DM)—a group of diseases with one of the highest incidence rates in the world, is defined by elevated blood sugar levels. People diagnosed with DM are more likely to experience a variety of life-threatening health issues, which can raise healthcare expenses, decrease quality of life, and even increase mortality rate [54]. Chronical high blood sugar levels harm all of the arteries, including those in heart, kidneys, and eyes. It is expected to affect 693 million people by 2045 [54], pointing out an increase of more than 50% from 2017.

People diagnosed with DM frequently develop chronic wounds due to the retarded wound healing process. As a result of this, they have a higher risk of experiencing frequent infections, as well as other severe problems [55]. Such a wound infection can spread to other tissues near the wound, eventually traveling to more distant body parts. An infection may be life-threatening in some situations if a patient does not receive immediate medical treatment. A diabetic patient’s chance of having a diabetic foot ulcer is 15–25%, and 40–80% of these ulcers progress to osteomyelitis, inflammation, or swelling in the body [56]. Patients who develop foot ulceration usually need hospitalization, surgical treatment, and sometimes even amputation of the affected part. A foot ulcer can be long-lasting, and patients are more likely to experience recurrence three years after the first appearance [57]. On account of this, poor wound healing in DM poses a serious problem and a considerable financial burden.

Different medications, mostly including dressings such as gels and creams, have been utilized to promote healing of diabetic wounds [58]. One of the advanced wound care products against diabetic wounds is using topical bandages, providing the moisture needed for healing and promoting the growth of fibrous tissue and epithelialization [58]. Additionally, topical bandages help to minimize scarring, enhance wound healing, and lower infection risk. Numerous types of dressings have been produced, and they all have some common characteristics, such as moisturization, stimulation of re-epithelialization, minimization of any possible trauma, and antibacterial qualities [59]. The other method is the use of antidiabetic drugs. Reports in the literature have shown that these drugs not only have an anti-inflammatory effect, but also play an important role in the production of macrophages that contribute to the healing of wounds, fibroblast proliferation, and keratinocyte proliferation [60]. It is also known that exogenous growth factors are used for the treatment of wounds [61]. A number of molecular and cellular reactions can be triggered by growth factors, which have the capacity to interfere at different phases of the recovery process. They are capable of stimulating angiogenesis, granulation tissue development, inflammation response regulation, extracellular matrix (ECM) production, and re-epithelization [61].

### 2.2. Skin Cancer

Skin cancer is an uncontrolled growth of skin cells that usually appears on skin that has been exposed to sunlight. However, this prevalent type of cancer could also develop on parts of the body that are not often exposed to the sun. Melanoma and nonmelanoma (primarily consisting of squamous cell carcinoma and basal cell carcinoma) are two main kinds of skin cancer. Briefly, melanoma is a type of skin cancer that occurs as a result of the breakdown of melanocyte cells, which are responsible for the production of melanin pigments [62]. Melanocyte cells in the body multiply uncontrollably and rapidly. There are cells called squamous cells in the middle and outer layers of the skin. In the squamous cell carcinoma type, squamous cells tend to proliferate uncontrollably. It is more likely to occur in people who primarily have sun exposure or have dark skin [63]. Basal cell carcinoma occurs in the basal cells of the epidermis, which is in the uppermost layer of the skin. The main reason is a direct body exposure to the sun and UV rays [63].

Surgical procedure, cryotherapy, chemotherapy, radiotherapy, topical field application (anticancer creams and gels), and immunotherapies are used as treatment methods for skin cancer. Despite the fact that removal of the tumor is an efficient strategy for skin cancer, surgical procedures can be deformed and require further skin grafts to repair the abnormalities. In this scenario, postsurgery treatment may include the use of wound dressings to stimulate the healing process and minimize bacterial infections and tumor reappearance [64].

### 2.3. Psoriasis

Psoriasis is a lifelong, systemic inflammatory condition that mostly affects the skin and joints [65]. It has a significant mental and emotional impact on patients that extends further than physical aspects of the disease, impacting well-being and social communication. Psoriasis has a number of complications, such as cancer and coronary heart disease. Skin biopsies are rarely necessary because the diagnosis is mostly clinical.

Psoriasis causes skin cells to grow quickly. A thick, scaly plaque may cause severe itching and discomfort for the patients [66]. Psoriasis comes in a variety of forms, relying on how the scales look and where they are on the body. The symptoms of psoriasis frequently increase in response to environmental factors. However, there is still no cure for these symptoms, and the recent advances in psoriasis therapies imply that they are now able to minimize the frequency and intensity of flares. The proper medicine is basically prescribed based on severity of the disease. First-line therapy for mild to moderate illness includes topical corticosteroids, vitamin D3 substitutes, and mixed products [65]. Physicians of primary care can easily initiate and prescribe topical therapy because they work successfully. Systemic therapy is frequently needed to treat moderate to severe psoriasis. Co-occurring conditions such as psoriatic arthritis need to be taken into consideration when choosing a treatment plan.

### 2.4. Microbial and Viral Infections

Infections of the skin and soft tissues caused by microorganisms and fungi are among the most common infections worldwide [67]. These lesions are caused by either primary inoculation or, frequently, disseminated infection. Although they are common in immunocompetent hosts, immunocompromised people are more vulnerable to these infections due to deficiencies in their innate or adaptive immune responses. Microbial infections that penetrate skin barriers might affect any of the three skin layers. In general, an infection on deeper layers of a skin is more dangerous than those of the surface layers [68]. A wound, particularly on a large area, might provide an opportunity for simple entry into the body. Opportunistic pathogens or those that become pathogenic as a result of a host disturbance (i.e., wound, immunodeficiency, sickness, or age), can grow inside the body by colonizing on nutrient-rich substances, such as necrotic or hypoxic tissue [69]. Microbial infections caused by multidrug resistance (MDR) are significant concerns that need to be addressed immediately. MDR bacteria impede wound healing since most wounds will acquire infections at some time. The ESKAPE bacterial group (*Enterococcus faecium, Staphylococcus aureus, Klebsiella pneumonia, Acinetobacter baumannii, Pseudomonas aeruginosa,* and *Enterobacter* species) includes the most opportunistic and MDR pathogens capable of colonizing wounds [70]. Moreover, *Staphylococcus aureus* and *Pseudomonas aeruginosa* can also form a biofilm on the wound and medical equipment, worsening the condition and making biofilm bacteria exceedingly difficult, if not impossible, to cure. This is because bacteria within biofilms are 100–1000 times more resistant to antimicrobial treatments, slowing the healing of infected wounds [71]. Topical antibiotic therapy is critical for the treatment of skin infections; nevertheless, antibiotic efficacy is frequently diminished due to insufficient local drug concentration, increasing antibiotic-resistant strains, biofilm development, or the drug’s inability to reach the site of action. As a result, treating these illnesses with high dosages of antibiotics may lead to a high incidence of side effects, probable allergic responses, patient annoyance, and an increased risk of antibiotic resistance development [72].

Different microorganisms (Helicobacter pylori, Streptococci, Staphylococci, Yersinia, Mycoplasma pneumoniae), viruses (Hepatitis virus, Norovirus, Parvovirus B19), and parasites (Giardia lamblia, Entamoeba spp., Anisakis simplex) have been also related with chronic urticaria. Infection-mediated autoimmune responses and molecular mimicry may be involved in these disorders [73]. Additionally, a massive proportion of the global population suffers from viral skin infections [74]. Cold sores, for example, typically affect the oral and perioral region and are caused by herpes simplex virus type-1 (HSV-1) and occasionally by HSV-2 as well; however, this predominantly affects the vaginal area. They are often characterized by pain and suffering as premonitory indications, as well as the emergence of papules or vesicles that burst and create scabs, which go off after a certain time, and lesions that endure mending in due course. Topical therapies consisting of heat/laser treatment, natural cures, photodynamic therapy, and antiviral drugs are available for symptomatic alleviation [75]. For another example, herpes zoster (shingles) often manifests as a localized, painful cutaneous eruption and is a frequent clinical concern, particularly among individuals over the age of 50 and immunocompromised patients. The reactivation of varicella-zoster virus infection causes herpes zoster, which causes a severe rash with blisters [76].

## 3. Dermal and Transdermal Drug Delivery and Design Parameters for Patches

Skin serves as a means for administering drugs through various routes. Two major routes of drug delivery through the skin are dermal and transdermal [77]. These paths for drug delivery have gained significant attention over the past decade due to their numerous benefits, such as improved patient compliance; physicochemical protection for various drugs; suitability for unconscious/vomiting patients; avoidance of first-pass metabolism, which increases the bioavailability of the drug; decreased frequency of dose administration; and reduced risk of toxic side effects [77]. The physico-chemical characteristics of the active components govern dermal drug transport to a great extent. Limited cutaneous penetration of the medication requires extreme polarity or stringent hydrophobicity, large molecular mass, the presence of ionizable functional groups, and their dissociation at the pathophysiological pH of the skin layers [78]. Dermal (topical) medication administration is used to describe localized activity with little systemic absorption to the diseased locations inside the skin. Transdermal drug delivery, on the other hand, involves the use of a patch that is applied to the skin. The patch contains the drug in a matrix or gel form, which is slowly released over time [79].

Drug delivery through dermal application is appealing because it increases patient compliance and quality of life [80]. Applying a dose form to a specific region of skin to produce a localized effect is known as topical medication distribution. It is often used to treat skin conditions such as eczema or psoriasis when a systemic distribution of the medication is not the primary goal of the treatment. Corticosteroids, antifungals, antivirals, antibiotics, antiseptics, local anesthetics, and antineoplastics are among the medications that are typically used topically [81].

Transdermal drug delivery systems are distinct from dermal drug delivery systems since it introduces medication into the bloodstream over the skin at a set and regulated rate [82]. This route is commonly used for the delivery of hormones, such as estrogen and testosterone, and for managing of chronic conditions, such as pain and smoking cessation. Transdermal drug delivery has the advantage of providing a steady and consistent level of the drug in the bloodstream, avoiding the fluctuation that can occur with oral or injectable administration. This is particularly useful for drugs that have a narrow therapeutic window or for conditions that require a constant level of medication.

The use of dermal and transdermal patches on skin to treat superficial skin disorders dates back to the oldest existing records of humankind. However, only in the late 20th century did their use become a common practice along with technological advancements in drug delivery systems [83]. Basically, a patch is a transferrable adhesive material placed on skin to deliver drugs with a tunable release rate and dosage to promote healing to an injured area [84]. They are usually noninvasive and painless drug delivery approaches. Up to date, various patches have been designed and manufactured for cosmetic and pharmaceutical application [85]. There are essential design criteria for manufacturing such patch-mediated delivery systems (Table 3). These patches are usually composed of a drug molecule, an adhesive, and polymer matrix.

Drugs can be administered as either dermal or transdermal approaches using patches. Dermal delivery usually refers to the process of the transport of drugs applied on the skin to deeper skin layers. On the other hand, transdermal delivery refers to the transport of drugs into the deeper skin layers, including their absorption by each layer, finally reaching the blood vessels in the dermis layer followed by joining the bloodstream. The purpose of the dermal and transdermal drug delivery processes differs according to the type of disease or injury and the treatment process required. While the dermal pathway is utilized for a local treatment, the transdermal pathway is used for the treatment of a specific disease by following a certain period of time [88]. One should bear in mind that not all drugs are convenient for patch delivery. The main criteria for optimum selection of drugs is the fact that drugs need to pose sufficient therapeutic attainability, cost-effectiveness, and ability of penetrating the skin layers with no safety issues. Their hydrophilic/hydrophobic nature, molecular weight, half-life, skin permeation coefficient, and concentration of drugs are the other parameters to be considered prior to patch design [89]. Another fundamental issue in patch development is adjusting the properties of adhesive material, as adhesive is directly linked to the efficacy of the patch system [90]. First and foremost, the adhesive should have the ability to form a strong bond with the skin surface under light-to-mild pressure. The resistance against shear adhesion and the force required to peel it off from the surface are other criteria to be taken into account for adhesive design [91]. Once these parameters are assessed vigilantly, the patch is expected to remain attached to the skin during a specific time period regardless of tangential stress induced by body movements or other factors such as sweating or dry skin.

Selection of polymeric material is expected to be the most critical step for formulating a patch-mediated drug delivery system. Polymers have the capacity to form film once crosslinked with specific agents. These polymers are usually dissolved in a suitable solvent together with drug molecules, and once the solvent is evaporated, a thin film is formed with drug molecules dispersed in polymer. The nature of polymers determines the drug release properties of patches; hence, polymers are selected accordingly [92]. For instance, the fabricated polymeric material must have a smooth surface, which is characterized by zero percent constriction as a flatness factor [93]. The designed polymeric system needs to have a sufficient endurance against folding and optimum tensile strength to determine if they are easily breakable [94]. Moisture content and uptake values are also critical in selecting a suitable polymeric system for patch design since they significantly impact their film-forming properties [95].

In some cases, the patch system is not sufficient to deliver drugs due to their low skin permeation coefficients—a parameter related to molecular weight of a drug. In this regard, MNs assembled on transdermal patches are employed to overcome individual limitations of patches. MN patches are basically composed of solid arrays measuring hundreds of microns in length [8]. The main reason for including MNs in patches is that they are capable of penetrating the stratum corneum, promoting easier uptake of drugs by inner skin layers. Additionally, the MN patch system allows effective delivery of high-molecular-weight molecules [96]. MNs can be classified as solid, hollow, coated, and dissolving MNs [97]. However, only coated and dissolving MNs are suitable for implementation in the patch system. Different from polymeric patches alone (noninvasive), MN-based patches are minimally invasive, yet still superior as they cause less pain, tissue damage, and skin inflammation compared to conventional hypodermic needles [68]. In the next section, we will explore dermal and transdermal patches, including MN-based patches, made of sustainable materials for a more ecofriendly drug delivery.

## 4. Sustainable Materials for Dermal and Transdermal Patches

Pharmaceutical technology is drawing more attention to transdermal and topical drug delivery strategies. When treating skin diseases, using medications topically rather than orally may boost their effectiveness. Polymers, whether natural or synthetic, make up the majority of the components utilized to create drug delivery patches and are regarded as the core of topical delivery. These patches are designed to provide a controlled release of the drug over an extended period of time, which can improve the efficacy and safety of the treatment.

Natural polymers, such as silk fibroin, keratin, alginate, hyaluronic acid, cellulose, gelatin, pectin, collagen and chitosan, are particularly useful in these applications due to their biocompatibility and ability to form strong, flexible bonds with the skin. The natural origin and biocompatibility of these polymers minimize the risk of skin irritation and adverse reactions, making them ideal for use in dermal applications. The natural polymers’ ability to absorb and retain drugs, along with their flexibility and conformability to skin, make them ideal for sustained drug release and improved therapeutic efficacy. These polymers also have unique mechanical, chemical, and biological properties that can be tailored for specific applications, providing versatility for different medical conditions. Natural polymers are derived from renewable sources and are biodegradable, making them an environmentally friendly alternative to synthetic polymers. Additionally, the use of natural polymers in transdermal patches eliminates the first-pass metabolism associated with oral administration, thereby increasing the bioavailability of the drug. Furthermore, the ability of natural polymers to form hydrogels, which are hydrophilic networks, enhances the stability and protection of drugs, improving their stability and performance. Overall, the use of natural polymers in transdermal or dermal patches is an important example of how these materials can be utilized to improve human health and well-being.

### 4.1. Silk Fibroin

Silk fibroin (SF) is a protein that has natural fibers and is found in nature as the raw material of spider webs and silk. It is composed of two main types of amino acid chains: beta-sheet and alpha-helix [98]. These chains are organized into a crystalline and an amorphous region, which give silk fibroin its unique properties. Typically, degumming, a technique for extracting silk gum from the silkworm *Bombyx mori*, is used to purify SF [99,100]. For centuries, SF has been regarded as a natural resource for the textile sector. On the other hand, in recent years, it has drawn significant interest as a potential biopolymer for biomedical practices because of its distinct mechanical and physicochemical characteristics. One of the notable characteristics of SF is its strength and toughness. The beta-sheet structure in the crystalline region offers strength to the SF structure, while the amorphous region gives durability [101]. Hence, this makes SF ideal for a wide range of applications, from clothing and textiles to biomedical industry. Another remarkable characteristic of SF is its biocompatibility [102]. SF is nontoxic and nonimmunogenic, making it a suitable material for use in medical implants and other biomedical applications. Additionally, SF has been shown to be biodegradable, which means it can be broken down by the body over time [103]. It also has favorable mechanical properties, making it a good candidate for use in biomedical engineering and other applications where strength and durability are important. Overall, due to its biocompatibility, easy processability, controllable biodegradation, versatile functionalization, and adjustable drug release qualities, SF has been frequently used to create drug delivery systems [104]. A variety of silk-based patches have been fabricated, such as silk-based microneedle (MN) patches that exhibit high biocompatibility and can entrap bioactive biomolecules inside the silk matrices before delivery [105].

**Figure 5:**
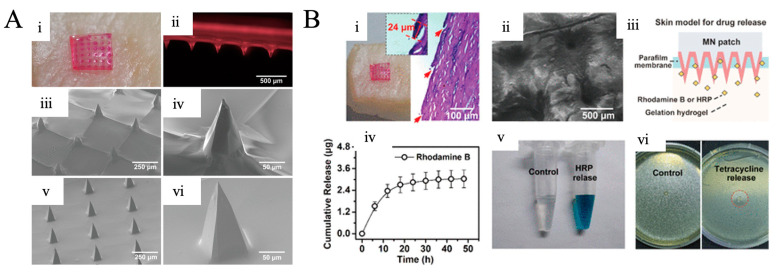
(**A**,**B**) Silk MN patches for transdermal delivery. (**A**) Silk fibroin microneedle patch morphology. (i) A drug-loaded silk fibroin MNP with Rhodamine B dyed covering pig skin. (ii) An image of SF MNP under the bright field. SEM images of (iii,iv) SF MNP and (v,vi) double layer SF MNP. (**B**) The two-layered SF MNP is used as drug carrier and is implanted into the skin. (i,ii) SF MN patch implemented to pig skin: (i) image and histological segment displaying the penetration sites of MNPs, and (ii) SEM image of pig skin that has been pierced. (iii) Schematical representation of HRP and Rhodamine B release from the skin model. (iv) The total amount of rhodamine B discharged from a SF MNP. (v) HRP bioactivity in a hydrogel produced from an SF MNP. (vi) Inhibition zone of *E. coli* with antibiotic-loaded SF MNPs. Reprinted with permission from [107], copyright 2021 American Chemical Society.

As an example from the literature, Rojas et al. developed SF-based polymeric MNs to increase the penetration ability of drugs [106]. This work used SF microneedles to administer porphyrins transdermally for possible use in photodynamic therapy (PDT), which combines a photosensitizing drug and visible light to kill abnormal cells. In this method, cytotoxic reactive oxygen species (ROS), which play an important role in cell death, are produced [106]. PDT is frequently used in combination with topical treatments, but the photosensitizers have demonstrated limitations in penetrating the skin layers. An ex vivo Franz diffusion cell was used to test the material’s cytotoxicity and transdermal transfer on pig skin. As a result of the study, it was seen that they increased the penetration of drugs with the silk fibroin-based MN patches they produced. This study was a pioneering study for photodynamic therapy, which is also used in the treatment of skin cancer. In another study reported by Yin et al., controlled tetracycline antibiotic release was performed using silk fibroin MN patches. MN patches produced using silk fibroin have been applied topically to the skin with an antibiotic drug and have been observed to reduce local infections [107] (Figure 5A). In the study, tetracycline-loaded silk fibroin MN was penetrated into pig skin and Rhodamine B release experiments were carried out. In addition, inhibition zone tests were performed for *E. coli* bacteria, and the drug release effect of the patches was demonstrated (Figure 5B). The method developed in this study has been a promising study in many wounds. Thanks to the high biocompatibility and controllable drug release properties of silk fibroin, the rapid healing of wounds without infection is a comfortable situation for many patients.

### 4.2. Chitosan/Chitin

Chitosan is produced from chitin with the employment of the deacetylation process. The quantity of amino groups and their water solubility are improved when chitin is partially deacetylated and transformed into chitosan. Moreover, the deacetylation process and biocompatibility and biodegradability have a proportional relationship in chitosan. Chitosan contains glucosamine and N-acetylglucosamine in its polysaccharide structure [108]. Considering its low toxicity, biocompatibility, and biodegradability, chitosan is widely employed in tissue-engineering applications [109]. In addition to wounds and cancer cells that form on the skin, the prevention and treatment of burns is also critical. Precautions taken for these burns and regular treatment processes are of great importance, especially in terms of preventing bacterial infection [110]. For instance, Boucard et al. fabricated a flexible chitosan-based hydrogel for healing of third-degree burns. Tulle Gras commercial product and viscous solution of chitosan was compared to the efficiency of chitosan hydrogels [111]. The amount of collagen I and collagen IV were evaluated to assess the extracellular matrix (ECM). The porous structure of physical hydrogel of chitosan provided the nutrients transportation through its pores and demonstrated a decrease of ~85% in scar surface in comparison to viscous solution and Tulle Gras, which exhibited a ~75 and 65% decrease, respectively. Chitosan polysaccharides, a biocompatible and natural polymer, can be used for important allergic conditions, as well as burns. For instance, Pavel et al. produced and tested chitosan bandages for shellfish allergies [112]. These bandages were used for this allergic function, which was observed in only 10 out of 40 participants. As a result, while no side effects were observed in seven people, slight side effects were observed in three people. In addition, the effects of using bandages against shellfish allergy were evaluated by measuring the IgE level, and positive results were observed in only 1.8% of the entire group. In addition to allergic studies, studies on delayed healing of wounds of diabetes patients are of great importance. Colobatiu et al. obtained streptozotocin (STZ)-loaded chitosan films in their study and used these films for diabetic wounds [113]. On the fourteenth day, the STZ-loaded chitosan film provided a healing rate of approximately 95%, while the reference (Betadine) and blank chitosan film provided a healing rate of approximately 60% and 88%, respectively. In a similar study, Hao et al. produced bio-multifunctional benzaldehyde-terminated 4-arm PEG (4-arm-PEG-CHO)/carboxymethyl chitosan (CMCS)/basic fibroblast growth factor (bFGF) hydrogels (BP/CS-bFGF) for diabetic wound repairing by boosting the Ki67 synthesis process, which increases the generation rate of collagen and epithelization. This hydrogel, with a 68.0 nm ± 21.3 nm porous structure, increased the healing process to an approximately 100% recovery rate after 14 days, while the control group and bFGF-free hydrogel (BP/CS) demonstrated approximately an 80% and 85% healing rate, respectively [114].

### 4.3. Alginate

**Figure 6:**
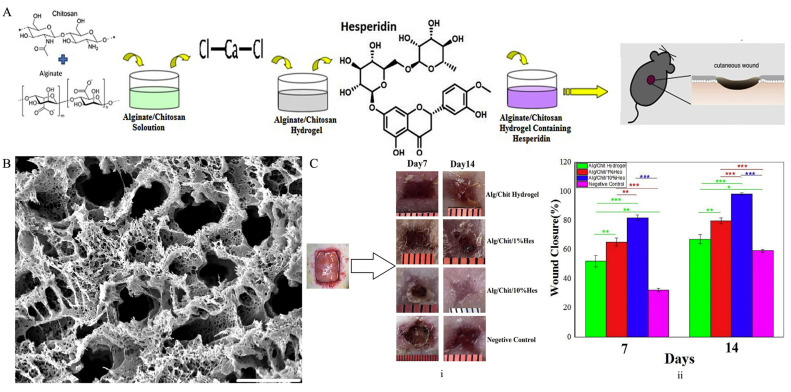
Wound repair with hesperidin-containing alginate/chitosan hydrogel. (**A**) Schematical representation of alginate/chitosan hydrogel preparation. (**B**) Alg/Chit/1%Hes under the SEM (Scale Bar: 100 μm). (**C**) (i) Wound morphology at 7 and 14 days after injury. (ii) Healing process at 7 and 14 days after injury. Values represent the mean ± SD, n = 6, * *p* < 0.05, ** *p* < 0.01, and *** *p* < 0.001. SD: standard deviation. Reprinted with permission from [122], copyright 2020 Elsevier.

Alginate is a significant tool for the drug delivery and tissue-engineering applications because it is biocompatible, biodegradable, and inexpensive and has easily producible advantages [115,116,117]. This natural linear polysaccharide is composed of D-mannuronic acid (M) and L-guluronic acid (G) in varying ratios and generated from brown algae or bacteria. Alginate’s high G block concentration allows for the production of rigid hydrogels containing divalent cations, such as Ca^2+^, and each of these cations binds to two opposing G blocks in an organized manner to create the so-called egg-box conformation [118]. In most cases, these polysaccharides are processed as nanofibers or film scaffolds with the loading of cancer therapeutics for the treatment of skin cancer [116,119]. For instance, Muthulakshmi et al. loaded the sodium alginate (SA) fibers with *Terminalia catappa* (TC) which is an effective antitumor medicinal plant [120]. Consequently, reactive oxygen species (ROS) generation and the detriment on the nuclear of B16F10 skin melanoma cancer line were boosted, and the cell growth was cut down from 100% growth without SA@TC application to approximately 35% growth with the 50 µg/mL concentration of SA@TC. On the other hand, Chiaoprakobkij et al. loaded bacterial cellulose/alginate/gelatin (BCAGG) stretchable biopolymer film with curcumin for the treatment of oral cancer cells (CAL-27) without damaging human keratinocytes (HaCaT) and human gingival fibroblasts (GF), which are healthy cells [121]. Cell viability tests demonstrated approximately 75%, 50%, and 40% cell viability of CAL-27 with the 4, 6, and 8 mg/mL curcumin solution concentration, respectively. In another study, the wound healing process was conducted using a rat model with the employment of hesperidin-loaded alginate/chitosan hydrogels [122] (Figure 6A–C). The nontoxicity of hesperidin-loaded hydrogel was confirmed using MTT assay with the observation of increase in cell proliferation. Wound closure was measured as approximately 60%, 65%, 80%, and 95% for negative control, alg/chit hydrogel, alg/chit/1%Hes, and alg/chit/10%Hes at the fourteenth day, respectively. In a related study, Cleetus et al. demonstrated that the ZnO nanoparticle-loaded alginate hydrogels performed an outstanding effect on the wound healing therapy with an increase of the percentage of cell in the injured area from 90% to approximately 96% after 24 h [123].

### 4.4. Keratin

When compared to certain other structural protein molecules, keratin, which is the central element of feathers, hooves, wool, horns, and hair, has a greater cysteine content (7–13%) [124]. It is composed of long chains of amino acids that are organized into alpha-helix and beta-pleated sheet structures [125]. These structures give keratin its strength and toughness qualities [126]. Another important characteristic of keratin is its water resistance [127]. Keratin fibers do not absorb water easily due to their structure, making them resistant to bending and breaking when wet. Keratin is also biocompatible and nontoxic, making it a suitable material for use in medical and cosmetic products, and it has found to be noninflammatory, nonallergic and nonimmunogenic [128]. Because of their outstanding biocompatibility, mechanic durability, easy availability, and biodegradability characteristics, keratin-based biomaterials have become the focus of significant research in the last few years in the fields of drug delivery, biomedical studies, and wound healing.

Nayak and Gupta, as an example, produced a keratin-based transdermal patch for the rapid healing of diabetic wounds [129]. They aimed to accelerate wound healing by loading glucose oxidase into these keratin patches because the excessive glucose concentration around the wound slows the healing process since glucose leads to hardened cell walls. Blood flow via arteries at the wound area ultimately becomes blocked. The flow and permeability of red blood cells, which are necessary for the growth of dermal tissue, are restricted by this effect [129]. As a result of this study, the potential applicability of keratin-based dermal patches for reducing topical glucose levels in diabetic wounds and especially foot ulcers has been demonstrated extensively.

On the other hand, malignant tumors are frequently treated with radiotherapy in addition to surgery. One concern is that the patient would have significant physical and psychological trauma as a result of the wound and improper wound healing condition [130]. When radiation is given in addition to wounds formed after surgical procedures, tissue formation and proliferation necessary for healing will be suppressed. For this reason, the healing time of the wounds will be prolonged. Based on this situation, Chen et al. observed that the healing of radiated-wounds accelerated with the keratin-based hydrogels they developed [130]. This study has provided significant steps for faster recovery for radiated-wound healing of skin cancer patients.

### 4.5. Gelatin

**Figure 7:**
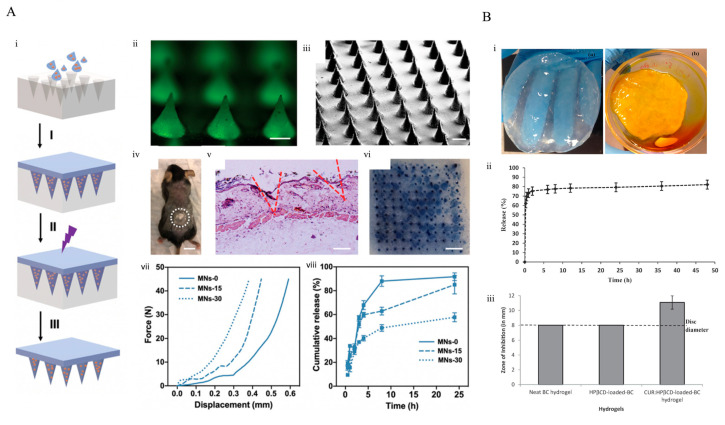
(**A**) Characterization of GelMA-β-CD based MNs. (i) Fabrication of MNs includes centrifugation, UV crosslinking and dry steps. (ii) Fluorescence display of MNs filled with curcimin which is visible with green fluorescence (Scale Bar: 200 μm). (iii) SEM visualization of MNs (Scale Bar: 300 μm). (iv) Transcutaneous application of the MN patch on a mouse, the closed dash circle represent the treatment site (Scale Bar: 1 cm). (v) A view of mouse skin stained with H&E demonstrating MN patch penetration (Scale Bar: 200 μm). (vi) MN patch penetration into the skin. Skin from a mouse cadaver was stained with 0.5% Trypan Blue after MN patch was attached (Scale Bar: 2 mm). (vii) Test of mechanical strength. (viii) Release test of curcimin. Reprinted with permission from [135], copyright 2020 Wiley. (**B**) Curcimin-loaded bacterial cellulose hydrogels. (i–a) Purified bacterial cellulose hydrogel. (i–b) Curcimin-loaded bacterial cellulose. (ii) A 48 h release profile from CUR:HPβCD-filled BC hydrogels. (iii) Disc diffusion test evaluation of antimicrobial efficacy against *S. aureus* for clean BC, HP-loaded-BC, and CUR:HP-loaded-BC hydrogels. Reprinted with permission from [143], copyright 2019 Elsevier.

Gelatin, a fibrous protein, is frequently used in tissue engineering studies due to its biocompatibility, biodegradability, and easy processing [131]. This protein, which is derived from collagen, has a variety of functional groups, including a set of ionizable groups, such as aspartic acid -COOH groups, terminal -NH2 and -COOH groups, the -NH2 group of lysine, the imidazolium group of histidine, guanidinium group of arginine, as well as carboxyl and phenolic groups, that can be utilized as potential sites for different chemical modifications. Owing to the cross-linkable and graftable groups, it exhibits good flexibility to adjustments [132,133]. Those types of modifiable polymers can be utilized purposefully in many applications. Tissue-engineering studies of gelatin range from scaffold function essential for cell culture to wound healing and cancer applications. For instance, since wounds take longer to heal for diabetics, the potentials of infection and inflammation are high. In order to carry out the healing process of this wound in a scar-free manner, sprayable gelatin methacrylate hydrogel was loaded with zeolitic imidazolate frameworks (ZIF-8) nanoparticles [134]. ZIF-8 itself produces HClO by consuming glucose and inhibits bacterial growth by reducing the glucose concentration in the wound. Bacteria viability of *Staphylococcus aureus* and *Escherichia coli* was reduced from approximately from 100% to ~20% and ~10% with the application of ZIF-8-loaded hydrogel, respectively. In another study, MN technology, which is frequently used, was employed to increase the release capacity and transdermal penetration of drugs. In this study, Zhou et al. aimed to treat B16F10 melanoma cancer cells by loading β-cyclodextrin into gelatin methacryloyl hydrogel and showing the efficacy using the release of curcumin (Figure 7A) [135]. These MN arrays (600 µm in height) were fabricated using UV crosslinking with 0, 15, and 30 s, and they were named as MNs-0, MNs-15, and MNs-30. Consequently, 50% of curcumin was released from MNs-0, whereas MNs-30 demonstrated higher efficiency with releasing 90% of curcumin. Furthermore, MNs-30 provided approximately 75% of cell viability, while MNs-0 and MNs-15 exhibited ~50% and ~60% of cell viability, respectively. In a related MN-based study for melanoma cancer treatment, Chen et al. fabricated MNs containing of a carboplatin (CP)-loaded gelatin, and their height was 600 μm. Viability in cancer cells dramatically decreased down to 40% with the implementation of 100 μM of CP loaded gelatin MNs after 72 h. Post-treatment results demonstrated that the CP-free MNs exhibited an anticancer effect on B16f10 cells, while the CP-loaded MNs exhibited better recovery results. CP-loaded MNs extended the survival period approximately from 12 to 20 days for the post-treatment process, while the CP-free MNs extended that process to 17 days [136].

### 4.6. Cellulose

One of the most prevalent biomaterials in the environment and the major component of plants is cellulose. It is a linear, unbranched polysaccharide that it is composed of repeating units of glucose. Furthermore, cellulose molecules are organized into microfibrils that are held together by hydrogen bonds [137]. Due to its linear and unbranched structure, cellulose molecules pack closely together, forming a dense network of hydrogen bonds between them. This gives cellulose its high tensile strength and stiffness [138]. Researchers have explored its use in various applications such as wound dressings, drug delivery vehicles, tissue-engineering scaffolds, biodegradable implants, and filtration membranes. The recent years have made it clear that bacteria can also make cellulose. Bacterial cellulose provides distinct mechanical and physical characteristics such as biocompatibility and purity, all of which encourage its use in the biomedical industry, and it also has a fibrous and tough structure [139,140]. Its water-insoluble structure allows cellulose to be used frequently in research. Many wound dressings have been produced, especially using bacterial cellulose. The release of different growth factors into the wound site plays a major role in the frequent use of cellulose in wound healing. Basic fibroblast growth factor, epidermal growth factor, and phosphodiesterase growth factor come to the wound area of dermal fibroblasts and ensure the proliferation of fibroblasts there. It also prevents the growth of bacteria in the wound [141]. Dermafill^TM^ product is produced for the healing of wounds after burns [142]. It protects the wound from contamination while increasing the concentration and distribution of nutrients and growth factors required for healing. Bionext^®^ is produced for skin ulcers (caused by diabetes, etc.) and burns [142]. Meanwhile, cellulose nanocrystals can be used as carriers for targeted drug delivery, helping to improve therapeutic outcomes [139]. Cellulose-based scaffolds are also being developed for tissue-engineering purposes, as they provide a physical support for cells to grow and differentiate into functional tissue [140].

For use as a topical application, Gupta et al. created bacterial cellulose hydrogels that were curcumin encapsulated [143]. (Figure 7B). The researchers indicated that the hydrogels can create a humid environment in the wound area based on the water vapor transmission capacity. Studies on the in vitro release of curcumin from cellulose-based hydrogels revealed that after 6 h, the loaded curcumin was released, accompanied by a slow and sustained release of the drug process [143]. In this way, the curcumin-loaded patch created on the wound can be used effectively in both microbial skin wounds and psoriasis-induced wounds.

For the appropriate and successful dermal treatment of psoriasis, Latif et al. fabricated methotrexate-loaded patches using a fusion of hydrophilic (hydroxypropyl methylcellulose—HPMC) and hydrophobic polymers (ethyl cellulose—EC), with enhanced dermal accumulation of methotrexate to improve its local effect [27]. The current investigation found that methotrexate with EC/HPMC polymers at different doses forms the best patches. All formed patches were tested, and the suitable patch formulation had the maximum methotrexate deposition and in vitro and ex vivo drug release pattern. Psoriasis affects the epidermis and dermis; thus, an increased retention on the deeper skin layers is key for the treatment. This EC/HPMC patch was ideal for transdermal medication administration. The controlled and gradual medication release made EC/HPMC patch composition appropriate for transdermal patches. Amorphous methotrexate was evenly dispersed in the patches [27].

### 4.7. Hyaluronic Acid

**Figure 8:**
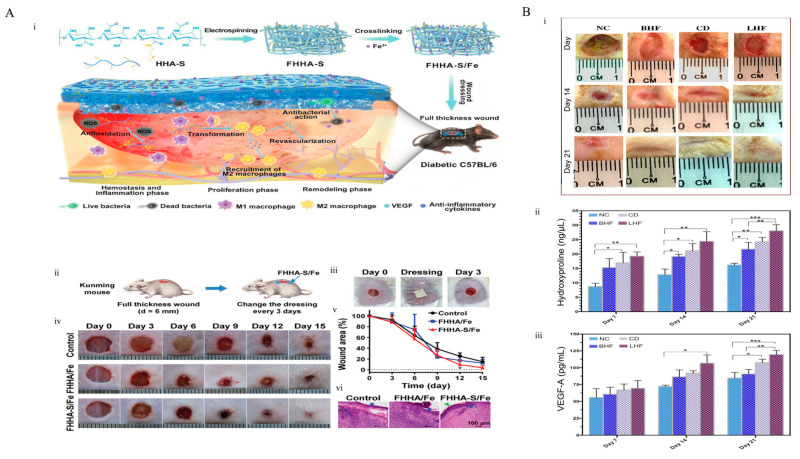
(**A**) Representation of hyaluronic acid nanofibrous hydrogel which grafted with thioether used for diabetic wound healing, (i) preparation of FHHA-s/Fe, dressing of hydrogel in diabetic mouse, action of mechanism of FHHA-s/Fe for chronic wound healing. (ii–vi) On an acute wound model, the FHHA-S/Fe nanofibrous hydrogel improved the healing. (ii) (**A**) The conceptual design and treatment of an acute wound. (iii) Display of Day 0 of the wound on the mouse, dressing with nanofiber, day 3 after treatment. (iv) Images of wounds following various treatments at the given days. (v) Quantitative results of the comparison of the initial wound with the indicated days. (vi) Pictures of the H&E-stained wound tissues at day 15. Reprinted with permission from [148], copyright 2020 Wiley. (**B**) Simvastatin loaded Alginate-Pectin hydrogel for treatment of diabetic wound. (i) Wound healing rate under various conditions. (ii) On days 7, 14, and 21, the levels of hydroxyproline various treatment cohort. (iii) The levels of VEGF-A in various treatment cohorts on days 7, 14, and 21. Values represent the mean ± SD, n = 6, * *p* < 0.05, ** *p* < 0.01, and *** *p* < 0.001. SD: standard deviation. Reprinted with permission from [149], copyright 2021 Elsevier.

Hyaluronic acid (HA), another polysaccharide, is frequently used in skin disease studies owing to its biodegradable and biocompatible properties [144]. In detail, HA is a naturally occurring polymer that is a member of the heteropolysaccharide class of glycosaminoglycans (GAGs), which are also present in the skin, connective tissue, joints, rooster comb, and umbilical cord of humans. Carboxyl and acetamido groups on its structure enable the formation of H-bonds with water molecules, stabilizing secondary structure in the process. This advantage provides biodegradability, especially in a water medium [145,146]. A methotrexate (MTX)-loaded MN patch was produced and applied by Du et al. for the treatment of psoriasis, one of these diseases [147]. The penetration capability increased owing to these arrays with a height of 650 µm and a base width of 220 µm, and this comparison was assessed between MTX-loaded MN and oral MTX treatment. While the epidermal thickness remained approximately 90 µm in oral treatment, this result was approximately 60 µm in MTX-loaded MN treatment. This shows us that the treatment with MN gives better results. On the other hand, HA has been utilized in many studies for the healing of wounds in diabetic patients. For instance, Liu et al. accelerated the healing process of chronic diabetic wound with the thioether-grafted HA nanofibrous hydrogel [148]. (Figure 8A). Reactive oxygen species (H_2_O_2_) were eliminated from the microenvironment and inflammatory reactions were reduced as a result of the thioether utilized. As a result, owing to this hydrogel employed, approximately 100% of the wound area was healed after 15 days, while hydrogels without thioether were able to heal 85% of the wound area in the same time.

### 4.8. Pectin

Pectin, one of many other natural polymers in nature, is frequently used in wound healing and tissue-engineering applications with its biocompatible and biodegradable properties [150]. Pectin—a heteropolysaccharide—has significant residues such as D-galacturonic acid (GalpA) in its chain structure. A key factor in controlling the solubility of pectin as well as its gelling and film-forming capabilities is the degree of esterification (D.E.) of these galacturonic acid residues. The controllability and modifiability of these polymers make them a crucial tool for tissue engineering studies [151]. For instance, Rezvanian et al. fabricated a simvastatin-loaded alginate-pectin hydrogel film for diabetic wound healing [149] (Figure 8B). They emphasized the importance of simvastatin by comparing this hydrogel film with a commercial dressing and blank hydrogel. Consequently, simvastatin-loaded hydrogel demonstrated a ~95% wound healing rate, whereas the simvastatin-free hydrogel and commercial dressing provided ~80% efficiency in 21 days. As seen in this study, natural polymers can be used in combination with many other natural polymers. In another study, Hasan et al. produced a hydrogel by combining pectin with alginate and hyaluronic acid and loaded clindamycin on this hydrogel [152]. Clindamycin (Cly)-loaded hydrogel was employed for the treatment of methicillin-resistant *Staphylococcus aureus* (MRSA)-infected wounds. Clindamycin was employed for its antibacterial property and protein synthesis-inhibiting capability. The effect of Cly on the reduction of bacterial viability was evaluated between Cly-free and Cly-loaded hydrogels using confocal microscopy. After 24 h, the bacterial viability for Cly-loaded hydrogels decreased from ~10^9^ to ~10^2^, whereas the viability remained at the same level in Cly-free hydrogel. In a cancer study, Gazzi et al. developed nanocapsule imiquimod-loaded pectin hydrogels (PEC-NCimiq) for SK-MEL-28 melanoma cell line. The nanocapsulation process of imiquimod enhanced the needed permeability to approximately 6 µg after 24 h, while imiquimod was permeated at about 4 µg in the nanocapsulation free process. Moreover, 7 µg of imiquimod penetrated to the dermis layer of skin from PEC-NCimiq, while PEC-imiq provided only 4 µg of imiquimod penetration. After 72 h, the viability of cells decreased dramatically down to ~50% with the implementation of PEC-NCimiq [153].

### 4.9. Collagen

Collagen is a fibrous protein that is the main structural component of skin, tendons, ligaments, and cartilage. It is composed of long chains of amino acids that are organized into triple helix structures [154]. This structure offers high tensile strength and resistance. Furthermore, collagen has the ability to form hydrogels [155]. Its fibers have a high water content, and when they are hydrated, they form a gel-like material. This property makes collagen an ideal material for wound healing and tissue engineering, since it can mimic the natural extracellular matrix of the body. Additionally, collagen is an important biomaterial because it can be used in medical (support wound healing), pharmacological, cosmetic, dental, and many other fields. Its features such as biocompatibility, flexibility, easy availability, mechanical strength, and biodegradability give collagen a versatile role [156]. These properties have increased the use of collagen patches in wound healing and drug delivery systems in recent years.

Drug-loaded collagen patches were used for psoriasis patients recently. It is very important for psoriasis patients to keep their skin moist, because the penetration required for the drugs to take effect will not be sufficient in a skin with a low moisture level. Terzopoulou and colleagues presented a study using collagen hydrogel patches with excellent swelling ability to provide adequate moist conditions for the treatment of psoriasis [157]. The researchers demonstrated remarkable results in preventing the proliferation of psoriatic keratinocytes and sustaining such suppression over time by coupling such qualities with the medication curcumin. One of the first stages was to embed curcumin in chitosan nanoparticles in order to increase its physical stability [157].

Moreover, diabetic foot ulcers are a common complication in people with diabetes. This situation is very uncomfortable for diabetic patients because wound healing is very slow. The most extensively employed form of modern technology, collagen, has been widely developed to address this pressing issue. Collagen type I (Col-I) is thought to be essential to recruit growth factors to the wound area and to start tissue repair and wound healing. Nevertheless, in the diabetic foot ulcers example, the skin is ulcerated, causing the extracellular matrix to be damaged, resulting in a decrease in tissue integrity and Col-I insufficiency [158]. Ulrich et al. reported rapid wound healing and a significant reduction in the size of the wounds in patients with diabetic foot ulcers by using collagen-based wound dressings in a study they carried out [159].

In summary, many natural polymers and hydrogels of some natural polymers are frequently used in patch making. The sources, advantages, and uses of the natural polymers described in this article are summarized in Table 4. Additionally, the drug/molecules and patch types used in the treatment of skin diseases mentioned in the review are summarized in Table 5.

## 5. Commercial Products

Owing to much research, how natural polymers can be used for skin diseases has been brought to the literature. It is of great importance that these hydrogels and patches can be used by people. In particular, the availability of tapes, dressings, or bandages can prevent rapid bleeding or infection [169]. In addition, it is very important for the environment that commercial products are produced from sustainable sources (Table 6). Synthetic bandages, which have been used for years, take a long time to decompose in nature, especially polyurethane (PU) bandages, which are non-biodegradable [170]. Many companies have introduced their products to overcome this situation. Aloe Vera adhesive bamboo bandages produced by Patch are used for burns and blisters [171]. It is of great importance that the product is eco- and vegan-friendly. Another product used for burns is Burn gel DRESSING produced by Qualicare [172]. On the other hand, Hydrocolloid thin dressings produced by 3M Tegaderm are used for ulcers, wounds, and burns [173]. The widespread use of these products in the future is of great importance in terms of both human health and the environment.

## 6. Conclusions and Future Perspectives

This review discusses a potential solution for treating skin diseases in an environmentally friendly manner. Diabetic wounds, skin cancer, psoriasis, and microbial infections are among the most common skin diseases. Patients with any skin condition experience psychological traumas as well as physical traumas. Therefore, the effects of these diseases on the skin should be treated as quickly as possible. Transdermal patches, which deliver medication through the skin, have become increasingly popular in recent years as a way to treat a variety of conditions. However, the materials currently used in the biomedical field are unsustainable synthetic-fossil-based or expensive naturally produced materials that are produced in limited numbers. On the other hand, this review suggests that ecofriendly transdermal patches, made with natural biomaterials and biodegradable polymers, could be a sustainable solution for treating skin diseases. These patches would be made with natural ingredients such as plant and animals extracts which have been shown to have anti-inflammatory and antimicrobial properties. In this direction, recently, many natural materials such as silk-fibroin, alginate, keratin, collagen, cellulose, and many other materials are frequently used in wound healing, drug delivery systems, and gene therapies. They would also be biodegradable, meaning they would break down naturally in the environment and not contribute to pollution. This review also highlights that ecofriendly transdermal patches would have additional benefits beyond just being more sustainable. Natural biomaterials used in the patches can have a more targeted and effective treatment as well as reducing the risk of side effects. It has been observed in studies that transdermal patches developed using sustainable biomaterials accelerate wound healing and increase the efficiency of drugs by controllable drug release. They would be also an affordable solution for people in developing countries. As highlighted in this review, innovative and arising renewable technologies for the synthesis of biomaterials for treatments of skin diseases can pave the way toward a prosperous and sustainable culture for the benefit of humanity. In addition, apart from the skin diseases mentioned in here, the patches developed and being developed will be used in the treatment of many other skin diseases while having additional benefits such as a targeted treatment, reduced risk of side effects, and affordable solution.

## Figures and Tables

**Table 1:** Advantages and disadvantages of transdermal and dermal drug delivery systems.

Advantages	Disadvantages	REF
Non- or minimally invasive approach	Possible local irritations	[11]
Controlled drug release rate	Low permeability of skin limits the penetration	[12]
Provides steady drug concentration in plasma	Limited number of drugs can be administered	[13,14]
Eliminates the first-pass effect	Patches can be uncomfortable to wear for long-term use	[15,16]
Easily application and removal from skin	External factors may prevent the patch from sticking to the skin	[17,18]
Improvement in bioavailability		[19]
Reduction in the frequency of dosing		[20]
Prolonged duration of action		[21]

**Table 2:** Advantages and disadvantages of natural and synthetic polymers used in dermal and transdermal administration of drugs [38,39,40,41,42,43].

Natural Polymers		Synthetic Polymers	
Advantages	Disadvantages	Advantages	Disadvantages
Low-cost production in fabricating skin patches	Expensive extraction methods	Higher controlled release of drugs due to consistent properties	Less environmentally friendly patches
Low adverse reaction on the skin	They can be biodegraded by microorganisms	Easily tailored into specific applications	More toxicity to the skin
Lower toxicity and less risk of allergic reaction	Susceptible to environmental conditions	Strong adhesion to skin due to high mechanical strength and flexibility	Possible allergic reactions
Enhanced skin penetration	Limited number of functional groups for chemical modifications	Resistant to degradation by enzymes and microorganisms	Not widely accepted by the general public due to concerns over safety
Increased the effectiveness of the drug	The quality of raw materials can vary, hence potentially affecting on the performance of the patch	Improved shelf-life of the patch	Higher risk of skin irritation
Renewable and high biocompatibility		Large-scale production with high purity and reproducibility	Less biodegradability may lead to environmental issues
Nontoxic and environmentally friendly			

**Table 3:** Critical parameters in designing the patch.

Patch Design		
Drug [84]	Polymer [86]	Adhesive [87]
Molecular weight	Tensile strength	Binding force with skin surface
Half-life	Endurance against folding	Resistance against shear adhesion
Skin permeation coefficient	Flatness	Force required to peel it off
Concentration	Moisture content	
Hydrophilic/hydrophobic nature	Solubility in different solvents	
	Film-forming capacity	

**Table 4:** Natural polymers for patches with their sources, advantages, and application area.

Sustainable Biomaterial	Source	Advantages	Application Type	Applications from Literature
**Silk Fibroin**	Silkworm (*Bombyx mori*)	Biocompatibility, easy processability, controllable biodegradation, versatile functionalization, adjustable drug release	Wound dressing, bone regeneration, drug delivery, gene therapy [160]	Silk fibroin MN patches for photodynamic therapy [106] Antibiotic release with Silk fibroin patches [107]
**Alginate**	Brown seaweed	Biocompatibility, biodegradability, inexpensive, easily producible	Wound dressing, tissue engineering, drug delivery, bone regeneration [161]	Antitumor agent-loaded alginate patches [120] Curcumin-loaded alginate stretchable biopolymer for oral cancer treatment [121] Hesperin loaded alginate hydrogels for wound healing [122]
**Keratin**	Feathers, hooves, wool, horns, and hair	Biocompatibility, mechanic durability, easy availability, biodegradability	Drug delivery, wound dressing, cosmetics, implant filler [162]	Glucose oxidase-loaded keratin transdermal patches for diabetic wounds [129] Keratin-based hydrogels for radiation-wound healing after skin cancer treatments [130]
**Gelatin**	Mammalian bone and hide	Cell recognition, biocompatibility, biodegradability, easy processing	Wound dressing, gene therapy, tissue engineering, drug delivery, intrinsic activity [163]	ZIF-8 nanoparticles-loaded sprayable gelatin methacrylate hydrogel for wound healing [134] β-cyclodextrin-loaded gelatin methacryloyl hydrogel for treatment of melanoma cancer cells [135]
**Collagen**	Bovine skin and tendons	Good cell recognition, biodegradability, flexibility, easy availability, mechanical strength, biocompatibility	3D printing, wound healing, cosmetics, drug delivery, dental applications [164]	Curcimin-loaded collagen hydrogel patches for the treatment of Psoriasis related wounds [157] Collagen-based wound dressing for diabetic foot ulcers [159]
**Cellulose**	Plants and bacteria	Biocompatibility, high purity, water in-soluble	Wound healing, drug delivery, tissue bioscafolds, medical implants [165]	Curcimin-loaded cellulose hydrogels for treatment of microbial skin wounds and psoriasis-induced wounds [143] Methotrexate-loaded cellulose patches for dermal treatment of psoriasis [27]
**Chitosan/Chitin**	Shells of crustaceans, insects and fungi	Biologically renewable, biodegradability, biocompatibility,	Stem cell technology, drug delivery, wound dressing, cosmetic, functional foods [166]	Chitosan-based hydrogel for healing of third-degree burns [111] Chitosan bandages for shellfish allergies [112] Streptozotocin (STZ)-loaded chitosan films for diabetic wounds [113]
**Hyaluronic Acid**	Soy-based foods	Biocompatibility, biodegradability, easily functionalized	Wound healing, DNA carrier, cosmetics, aesthetic [167]	Methotrexate (MTX)-loaded hyaluronic acid MN patch for the treatment of psoriasis [147] Thioether-grafted hyaluronic acid nanofibrous hydrogel for healing process of chronic diabetic wound [148]
**Pectin**	Apple, citrus fruits, sugar beets	Biocompatibility, biodegradability	Tissue engineering, drug delivery, wound healing [168]	Simvastatin-loaded alginate-pectin hydrogel film for diabetic wound healing [149] Clindamycin-loaded pectin/alginate/hyaluronic acid hydrogel for the treatment of infected wound [152]

**Table 5:** Drug/molecules and patch types used in the treatment of skin diseases.

Drugs/Molecules	Patch Type	Application Purpose	Model Skin/Organism	Highlights	Ref
**Porphyrins**	Silk Fibroin Microneedle	Skin Cancer Treatment	Ex vivo pig skin	Enhanced the penetration of drugs, pioneering study for photodynamic therapy.	[106]
**Tetracycline Antibiotics**		Reducing Local Infections	Escherichia coli	Rapid healing of wounds without infection	[107]
**Streptozotocin (STZ)**	Chitosan Film	Diabetic Wound Treatment	Rat skin	Streptozotocin increased healing rate from 60% to 95% after 14 days.	[113]
**Basic fibroblast growth factor (bFGF)**	Chitosan Hydrogel		Mice Skin	Nutrition support with porous structure, healing enhancement from 80% to 100% after 14 days.	[114]
**Terminalia catappa (TC)**	Sodium Alginate Fibers	Skin Cancer Treatment	Skin melanoma cell line (B16F10)	Cell growth rate of cancer cells decreased from 100% to 35%.	[120]
**Curcumin**	Cellulose/alginate/gelatin (BCAGG) film	Oral Cancer Treatment	Oral cancer cells (CAL-27)	Stretchable film provided easy-to-use advantage, Cell viability reduced from 100% to 40% without damaging human keratinocytes (HaCaT) and human gingival fibroblasts (GF), which are healthy cells.	[121]
**Hesperidin**	Alginate hydrogel	Wound Treatment	Rat skin	Wound repairing rate was improved from 60% to 95% after 14 days.	[122]
**Zinc Oxide (ZnO) Nanoparticles**			CF-1 MEF IRR 2M mammalian fibroblast cells	Enhancing wound treatment from 90% to 96% after 24 h with suppressing bacterial growth.	[123]
**Glucose Oxidase**	Keratin transdermal patch	Diabetic Wound Treatment	Human	The potential applicability of keratin-based dermal patches for reducing topical glucose level in diabetic wounds and especially foot ulcers.	[129]
Keratin	Keratin Based	Skin cancer treatment	HaCaT keratinocytes and rats	Faster recovery for radiated-wound healing.	[130]
**Zeolitic imidazolate frameworks (ZIF-8) nanoparticles**	Sprayable gelatin methacrylate hydrogel	Wound Treatment	Staphylococcus aureus and Escherichia coli	Decreasing viability of bacteria from 100% to approximately 20%.	[134]
**β-cyclodextrin**	Gelatin microneedle	Skin Cancer Treatment	Skin melanoma cell line (B16F10)	Increase in the release capacity and transdermal penetration of drugs, advancement in the release of drug with extending curing process of gelatin microneedle.	[135]
**Carboplatin (CP)**				Cancer cell viability dramatically decreased from 100% to 40% after 72 h.	[136]
**Curcumin**	Cellulose hydrogel	Microbial skin wounds and psoriasis-induced wounds treatment	A549, U251MG, MSTO and Panc1 adenocarcinoma cell lines	Increase in the humid environment with the porous structure of hydrogel and obtained slow and sustainable drug release capacity.	[143]
**Methotrexate**	Ethyl cellulose—hydrophilic hydroxypropyl methylcellulose (EC/HMPC) patch	Psoriasis Treatment	Albino rabbits	Drug was distributed uniformly, and its local effect increased.	[27]
	Hyaluronic acid (HA) microneedle		Mice Skin	Reducing required treatment thickness from 90 µm to 60 µm.	[147]
**Thioether grafting**	Hyaluronic acid (HA) nanofibrous hydrogel	Diabetic Wound Treatment		100% healing rate after 15 days in comparison to hydrogels without thioether, which demonstrated 85% healing rate. Elimination of reactive oxygen species (H2O2) and preventing inflammatory reactions with thioether application.	[148]
**Simvastatin**	Alginate-Pectin hydrogel		Rat skin	Enhancing healing rate from 80% to 95% after 21 days.	[149]
**Clindamycin (Cly)**	Pectin hydrogel	Methicillin-resistant Staphylococcus aureus (MRSA)-infected wounds	Methicillin-resistant Staphylococcus aureus (MRSA)	Cell viability of bacteria decreased from 10^9^ to 10^2^.	[152]
**Nanocapsule imiquimod**		Skin Cancer Treatment	SK-MEL-28 melanoma cell line	Advancement in permeability of imiquimod with nanocapsulation process. After 72 h, the viability of cells decreased dramatically from 100% to approximately 50%.	[153]
**Curcumin**	Collagen hydrogel	Psoriasis Treatment	Keratinocytes and fibroblasts from the skin of Caucasian patient	Preventing the proliferation of psoriatic keratinocytes and sustaining such suppression over time.	[157]
**Collagen type I**	Collagen patch	Diabetic foot ulcer treatment	Human	Rapid wound healing and a significant reduction in the size of the wounds in patients.	[159]

**Table 6:** Sustainable Commercial Products.

Company	Active Material	Application	Highlights	Ref
Patch	Aloe Vera	Burns and blisters	Vegan product, sustainable, biocompatible, immediate applicability, and easy-to-use	[171]
Qualicare	Hydrogel	Burns and scalds	First-aid gel, nonadherent, sterile, biocompatible, and easy-to-use	[172]
3M Tegaderm	Hydrocolloid Thin Dressing	Ulcers, wounds, and burns	Enhanced exudate control, offered in sacral, square, and oval-shaped dressings, and waterproof film	[173]

## Data Availability

Not applicable.
